# Comparable outcomes for β-lactam/β-lactamase inhibitor combinations and carbapenems in definitive treatment of bloodstream infections caused by cefotaxime-resistant *Escherichia coli* or *Klebsiella pneumoniae*

**DOI:** 10.1186/s13756-015-0055-6

**Published:** 2015-05-01

**Authors:** Patrick N A Harris, Mo Yin, Roland Jureen, Jonathan Chew, Jaminah Ali, Stuart Paynter, David L Paterson, Paul A Tambyah

**Affiliations:** University of Queensland Centre for Clinical Research, Brisbane, Queensland Australia; Department of Infectious Diseases, National University Hospital, Singapore, Singapore; Yong Loo Lin School of Medicine, National University of Singapore, Singapore, Singapore; Department of Laboratory Medicine, National University Hospital, Singapore, Singapore; International Medical University, Kuala Lumpur, Malaysia; School of Population Health, University of Queensland, Brisbane, QLD Australia

**Keywords:** Extended-spectrum β-lactamase, *Enterobacteriaceae*, Piperacillin-tazobactam, Carbapenem

## Abstract

**Background:**

Extended-spectrum β-lactamase (ESBL) producing Enterobacteriaceae are often susceptible in vitro to β-lactam/β-lactamase inhibitor (BLBLI) combination antibiotics, but their use has been limited by concerns of clinical inefficacy. We aimed to compare outcomes between patients treated with BLBLIs and carbapenems for bloodstream infection (BSI) caused by cefotaxime non-susceptible (likely ESBL- or AmpC β-lactamase-producing) *Escherichia coli* and *Klebsiella pneumoniae*.

**Methods:**

All adult patients with a BSI caused by cefotaxime non-susceptible *E. coli* or *K. pneumoniae* were included from May 2012-May 2013. We compared outcomes between patients who had definitive monotherapy with a carbapenem to those who had definitive monotherapy with a BLBLI.

**Results:**

There were 92 BSIs that fulfilled the microbiological inclusion criteria. 79 (85.9%) were caused by *E. coli* and 13 (14.1%) by *K. pneumoniae*. Four out of 23 (17.4%) patients treated with carbapenem monotherapy and 2 out of 24 (8.3%) patients treated with BLBLI monotherapy died (adjusted HR for survival 0.91, 95% CI 0.13 to 6.28; p = 0.92). The time to resolution of systemic inflammatory response syndrome (SIRS) criteria did not vary between the treatment groups (adjusted HR 0.91, 95% CI 0.32 to 2.59; p = 0.97). The length of hospital admission post-positive blood culture was slightly longer in patients treated with BLBLIs (median duration 15 vs. 11 days), although this was not significant (adjusted HR 0.62; 95% CI 0.27 to 1.42; p = 0.26). There were no significant differences in subsequent isolation of carbapenem resistant organisms (4.3% vs. 4.2%, p = 1.0), *C. difficile* infection (13.0% vs. 8.3%, p = 0.67) or relapsed BSI (0% vs. 2%, p = 0.23).

**Conclusions:**

BLBLIs appear to have a similar efficacy to carbapenems in the treatment of cefotaxime-resistant *E. coli* and *K. pneumoniae* bloodstream infections. Directed therapy with a BLBLI, when susceptibility is proven, may represent an appropriate carbapenem-sparing option.

**Electronic supplementary material:**

The online version of this article (doi:10.1186/s13756-015-0055-6) contains supplementary material, which is available to authorized users.

## Introduction

Gram-negative bacteria that possess extended-spectrum β-lactamase (ESBL) enzymes have emerged as a major global public health concern in recent years [1]. When first recognized, these resistant isolates were usually implicated in nosocomially-acquired infections or outbreaks [2]. Today ESBL-producers are commonplace in the community [3,4] or in the broader healthcare context, such as residential aged-care facilities [5,6]. The burden of disease is particularly marked in the Asian region [7,8]. In Singapore, approximately 20% of *E. coli* and 32% of *Klebsiella* spp*.* are non-susceptible to third-generation cephalosporins [9].

Carbapenems have been regarded as the treatment of choice for serious infections caused by ESBL-producers [10,11]. However, the increasing worldwide incidence of ESBL-related infections is driving increased use of carbapenems, leading to selection pressure for carbapenem resistance [12,13]. Even brief exposure to a carbapenem can increase the risk of colonisation or infection with a carbapenem-resistant organism [14]. We now face the challenge of emerging carbapenem resistance, largely mediated by the efficient spread of carbapenemases in key Gram-negative pathogens [15,16]. Genes coding for carbapenemases are usually co-located with multiple acquired resistance determinants, leaving few effective, low toxicity or non-parenteral options for therapy [17].

In the face of this rapidly changing epidemiology, there is a pressing need to reduce carbapenem overuse. One strategy could be re-evaluating existing agents which have previously been considered ineffective or lack clinical data to support their use. The β-lactam/β-lactamase inhibitor (BLBLI) combination antibiotics, such as amoxicillin-clavulanate, ticarcillin-clavulanate and piperacillin-tazobactam, have a controversial status in the treatment of infections caused by ESBL-producers [1]. By definition, Ambler class A ESBL enzymes are inhibited by clavulanate or tazobactam in vitro and ESBL producers, especially *E. coli*, are frequently susceptible to BLBLIs. Yet, there have been concerns that such in vitro susceptibility may not reliably translate into clinical efficacy [18]. This has been based largely on concerns over inoculum effects, the co-location of other beta-lactamase enzymes (which may not be well inhibited by β-lactamase inhibitors) on acquired plasmids and the potential for additional resistance mechanisms such as alterations in outer membrane proteins [19].

Recent studies suggest that in certain situations, BLBLIs are non-inferior to carbapenems if isolates are susceptible in vitro, [20] especially if the minimum inhibitory concentration (MIC) is low [21]. Such a strategy may represent a reasonable carbapenem-sparing option for treating infections caused by ESBL-producers [19]. This study aimed to examine current treatment strategies for bacteraemia caused by cefotaxime non-susceptible *Escherichia coli* or *Klebsiella* spp. in an institution with a relatively high incidence of ESBL-producing isolates. Increasingly, BLBLIs are being considered for treatment of these infections as part of antimicrobial stewardship and carbapenem restriction. As such, we aimed to compare clinical and microbiological outcomes between patients receiving definitive carbapenem therapy with those given piperacillin-tazobactam, as a carbapenem-sparing treatment option.

## Materials and Methods

The study has been presented in accordance with the STROBE guidelines on the reporting of observational studies [22].

### Objectives

The aim of the study was to compare the efficacy of BLBLI antibiotics to carbapenems for the treatment of bloodstream infections caused by cefotaxime-resistant (likely ESBL- or AmpC-producing) *E. coli* or *K. pneumoniae*. The hypothesis was that there are no significant differences in outcomes for patients treated with a BLBLI or a carbapenem for bloodstream infections caused by cefotaxime-resistant *E. coli* or *K. pneumoniae*, if these isolates remain susceptible to piperacillin-tazobactam and meropenem in vitro.

### Design

A retrospective observational study examining a cohort of patients with bloodstream infection to compare outcomes following different antibiotic treatments.

### Setting

Patients were identified from admissions to National University Hospital (NUH), a tertiary referral hospital in Singapore with approximately 1,068 beds. The hospital also treats many patients from elsewhere in Asia and the Middle-East. The prevalence of ESBL-producing isolates is relatively high in patients presenting both from the community and with hospital-acquired infection.

### Ethics

The Domain Specific Review Board (DSRB) of the National Healthcare Group (NHG) in Singapore provided ethics approval for this study (NHG DSRB Ref: 2013/00877).

### Inclusion and exclusion criteria

Any adult patient (≥21 years) with a bloodstream infection due to *E. coli* or *Klebsiella* spp. identified through the NUH microbiology laboratory, defined by at least one monomicrobial positive blood culture between May 2012 to May 2013, was eligible for inclusion. Bacterial isolates were confirmed as cefotaxime non-susceptible, but piperacillin-tazobactam and meropenem susceptible by EUCAST standards [23]. Patients were excluded if they showed polymicrobial bacteraemia or if no antimicrobial therapy was given.

### Data collection

Data were obtained by clinical chart review and interrogation of the electronic prescribing and laboratory information systems. A standard clinical record form was used. Demographic information such as age, date of hospital admission and ethnicity were recorded. Community, hospital and healthcare acquisition of bloodstream infection was defined according to standard criteria (defined below) [24]. Concurrent co-morbidities defined at admission were recorded (see Additional file 1). The presence of a medical device in the 7 days prior to bloodstream infection was recorded; this included vascular catheters (peripherally inserted central catheter [PICC], dialysis catheter, central venous catheter or implanted line), central nervous system devices (e.g. external ventricular drain) and urinary catheters or nephrostomy tubes. Relevant therapy in the 30 days prior to bloodstream infection was documented, including cytotoxic chemotherapy, systemic corticosteroids (>15 mg of prednisolone daily or equivalent), anti-TNF (tumour necrosis factor) therapy in last 12 months, other monoclonal antibody therapy, use of immune suppressive agents or radiation therapy. Any surgery in 14 days prior to first blood culture was also recorded. All enrolled patients were stratified using the Charlson co-morbidity index (CCI) using pre-specified definitions (see supplementary material) [25]. Patients admitted to the intensive care unit (ICU) were also assessed using APACHE II scores [26]. Pitt bacteraemia scores were calculated based on clinical parameters measured within 24 hours of initial blood culture collection [27].

The probable source of bacteraemia was assessed according to the available clinical and microbiological information and classified by the investigators using the following categories: urinary tract, central nervous system, pneumonia, intra-abdominal, hepato-biliary, mucositis, line related, musculoskeletal, skin and soft tissue (including burns), surgical site infection, neutropenic sepsis, other or unknown source.

### Acquisition status

Acquisition status was defined as follows, in accordance with standard criteria [24]:

1. Hospital-acquired infection (HAI): afebrile on admission, blood culture positive >48 h after admission or within 48 h after discharge.
2. Community onset: blood culture collected <48 h after admission, not admitted or >48 h after discharge; then defined as healthcare associated if any of the following:
  - Hospitalisation (excluding natural birth) for 2 or more days within 90 days or attendance at emergency department within 2–30 days before bloodstream infection
  - Admission to an out-patient intravenous therapy or hospital-in-the-home service within 2–30 days before bloodstream infection
  - Chronic renal dialysis
  - Residence in long-term care facility
3. Community associated: none of the above

### Antibiotic use

Therapy was defined as empirical if the first dose was given within the first 72 hours following blood culture collection, before results of blood cultures were available (including susceptibility testing); definitive therapy was defined if initiated > 72 h following initial blood culture collection and for ≥50% of the total treatment duration. All Gram-negative active agents administered were recorded, including doses, frequency and duration in days. Prescribing data were collected using the hospital electronic prescribing system. Adequate empirical therapy was defined as the receipt of at least one parenteral agent (or oral agent with good bioavailability, such as a fluoroquinolone) to which the isolate was subsequently found to be susceptible in vitro. Intravenous doses of piperacillin-tazobactam 4.5 g 6-hourly or 8-hourly, amoxicillin-clavulanate 1.2 g 8-hourly, meropenem 1 g 8-hourly, ertapenem 1 g 24-hourly or imipenem 500 mg 6-hourly were used (with adjustment for renal dysfunction) as per local prescribing guidelines.

### Vital sign measurement

Maximum and minimum temperatures (T_max_ and T_min_), maximal heart rate, maximal respiratory rate, highest total white cell count (if tested), lowest systolic blood pressure, use of a vasopressor agent and results of any repeat blood cultures (if collected) were recorded daily for seven days.

### Outcome measures

1. Days to resolution of systemic inflammatory response syndrome (SIRS). SIRS was defined as being present if ≥2 of the following were recorded:
  - T_max_ >38°C or T_min_ < 36°C
  - Lowest systolic blood pressure <90 mmHg and/or inotrope requirement,
  - Respiratory rate >20/min or arterial PaCO_2_ < 32 mm Hg if ventilated,
  - Maximal heart rate >90/min,
  - Total white cell count >12 × 10^9^/L or <4 × 10^9^/L
  Days to resolution of SIRS was calculated from the date of initial blood culture collection to the first day where the patients did not fulfil SIRS criteria (as defined above). Patients were assumed to have resolution of SIRS on the date of discharge (if they did not have resolution of SIRS previously using the above criteria). Patients who died were included in the analysis, and defined as never having resolution of SIRS. Where daily white cell count values were missing, the value was imputed by calculating the midpoint difference between the adjacent day values. Patients for whom SIRS criteria could not be ascertained due to missing data were excluded from this part of the analysis.
2. All-cause mortality at 30 days post initial positive blood culture.
3. Identification of a carbapenem or piperacillin-tazobactam resistant isolate in subsequent 30 days or identification of *Clostridium difficile* infection.
4. Microbiological relapse (positive blood culture >72 h after initiation of definitive therapy and up to 30 days) with same organism as original initial blood culture.
5. Length of hospital stay post first positive blood culture. Patients who died were excluded from this analysis.

### Microbiological testing

Blood cultures were inoculated into BacT/Alert bottles (BioMerieux; Marcy-L’Étiole, France) and incubated for up to 5 days. Positive cultures were sub-cultured and identified to species level by standard laboratory methods, including MALDI-TOF (Bruker Daltoniks GmHB; Bremen, Germany) and Vitek2 (BioMerieux). Susceptibility testing was performed using Vitek2 microbroth dilution according to EUCAST interpretative standards [23]. Phenotypic testing or molecular methods were not routinely used to confirm ESBL or plasmid-mediated AmpC beta-lactamase production. Antibiotic reporting was at the discretion of the duty microbiologist at the time of the bloodstream infection. All positive blood cultures reported within the 30-day follow-up period were also recorded.

### Statistical analysis

Categorical data were presented as proportions and scale data using median and interquartile ranges. Patients who received carbapenems or BLBLIs as definitive monotherapy were identified and compared. Groups given other non-carbapenem or non-BLBLI therapy and combination therapy were excluded. Survival curves for mortality, days to resolution of SIRS, and length of hospital stay were presented using Kaplan-Meier curves. Cox proportional hazards regression was used to assess potential confounders identified *a priori* (age, acquisition status, Pitt bacteraemia score, ICU admission, organism, and whether or not the patient had received appropriate empirical therapy). Potential confounders were included in the final regression model if they caused a change in the main effect (hazard ratio according to definitive treatment) by 5% or more when included in bivariate analysis with definitive treatment. Statistical analysis was performed using Stata (StataCorp; Texas, USA).

## Results

**Figure 1:**
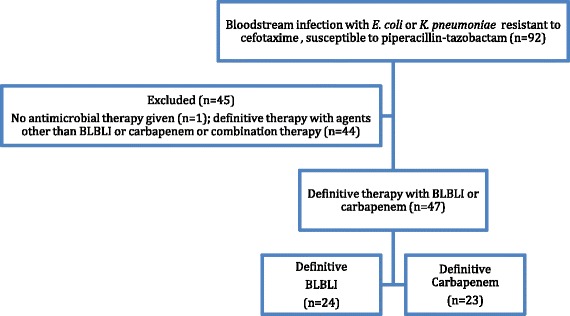
Patient inclusion flowchart - treatment with BLBLI or carbapenem.

**Figure 2:**
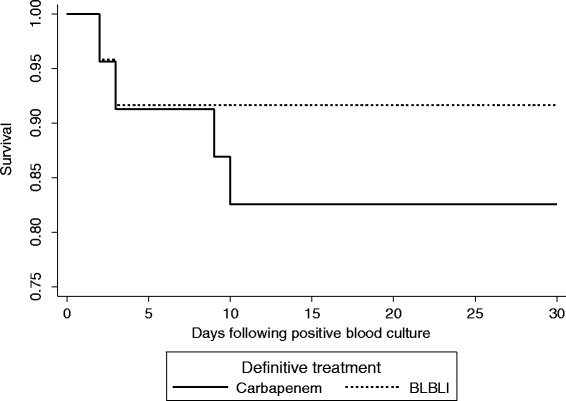
30-day mortality for patients treated with BLBLI or carbapenem as definitive monotherapy.

**Figure 3:**
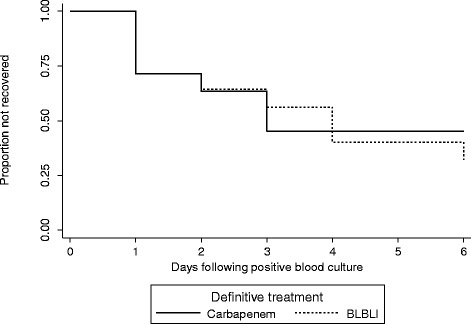
Days to recovery from SIRS (<2 SIRS criteria or discharge).

**Figure 4:**
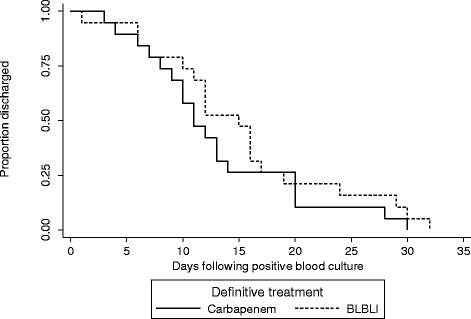
Length of hospital admission post-positive blood culture for patients treated with BLBLI or carbapenem as definitive monotherapy.

During the period from May 2012 to May 2013 there were there were 476 *E. coli* and 328 *K. pneumoniae* bloodstream infections in patients > =21 years old. Out of the total 804 bloodstream infection events, there were 92 (11.4%) that fulfilled the microbiological inclusion criteria, of which 79 (85.9%) were caused by *E. coli* and 13 (14.1%) by *K. pneumoniae*. Susceptibility patterns are summarized in Table 1 and MIC distributions for amoxicillin-clavulanate, piperacillin-tazobactam and cefoxitin are shown in Table 2. One patient was excluded as they were discharged without any antibiotic treatment. The median age was 75 years (range 23–100 years; IQR 21) and 53.8% were female. Of the 91 patients given treatment, 44 received definitive treatment that did not include a BLBLI or a carbapenem or were given combination definitive therapy, leaving 47 patients eligible for inclusion in the analysis for definitive therapy (24 receiving a BLBLI and 23 a carbapenem) (see Figure 1). Definitive carbapenem monotherapy included 60.9% given meropenem, 34.8% imipenem and 4.3% ertapenem; for definitive monotherapy with a BLBLI, 95.8% were given piperacillin-tazobactam and 4.2% amoxicillin-clavulanate. In the carbapenem treated group, 17.4% received ‘step-down’ once daily therapy with ertapenem following initial therapy with imipenem or meropenem. In the BLBLI treated group, 8.3% were given ‘step-down’ therapy with amoxicillin-clavualante following piperacillin-tazobactam. Other antibiotic choices, along with the baseline characteristics are reported in Table 3. Mortality was relatively infrequent: four out of 23 (17.4%) patients treated with carbapenem monotherapy and 2 out of 24 (8.3%) patients treated with BLBLI monotherapy (Figure 2). On univariate analysis of mortality, a non-significant result favouring BLBLIs was seen, but this was diminished after adjustment for confounders (see Table 4). The HR for survival at 30 days was 0.91 (95% CI 0.13 to 6.28; p = 0.92) after adjustment for ICU admission, infecting organism, and Pitt score. There was no difference in the time to resolution of SIRS between the two definitive treatment groups (HR 0.91, 95% CI 0.32 to 2.59; p = 0.97) (Figure 3 and Table 4). For the analysis of the length of hospital stay post positive blood culture, four patients were excluded because they had prolonged hospital admission (>40 days) due to unrelated factors. The length of hospital stay post positive blood culture was slightly longer in patients treated with BLBLI compared to carbapenem. For those treated with a BLBLI, the median length of stay was 15 days [IQR 10 to 19 days] while for those given a carbapenem, the median length of stay was 11 days [IQR 8 to 20 days]. In the Cox regression analysis, this difference was not statistically significant (HR 0.62, 95% CI 0.27 to 1.42; p =0.26) (Figure 4 and Table 4). There were no significant differences in subsequent isolation of a carbapenem resistant organism (4.3% vs. 4.2%, p = 1.0), *C. difficile* infection (13.0% vs. 8.3%, p = 0.67) or relapsed bloodstream infection (0% vs. 2%, p = 0.23).

**Table 1:** **Susceptibility profiles of** ***E. coli*** **or** ***K. pneumoniae*** **isolated from blood cultures with resistance to ceftriaxone during study period**

***Susceptibility category***								**Antimicrobial agent tested**									
***E. coli***	**AMP**	**AMC**	**TZP**	**CXM**	**CRO**	**CTX**	**CAZ**	**FEP**	**FOX**	**IPM**	**MEM**	**ETP**	**LVX**	**CIP**	**SXT**	**GEN**	**AMK**
R (%)	100	60	0	100	100	100	96	0	11	0	0	0	71	71	59	35	0
I (%)	0	0	0	0	0	0	4	4	20	0	0	0	0	3	0	0	3
S (%)	0	40	100	0	0	0	0	96	68	100	100	100	29	27	41	65	97
Total tested	79	73	79	79	79	79	79	79	79	79	79	79	79	79	79	79	79
***K. pneumoniae***																	
R (%)	100	45	0	100	100	100	100	92	0	0	0	0	15	30	85	31	0
I (%)	0	0	0	0	0	0	0	8	8	0	0	0	0	40	0	0	0
S (%)	0	55	100	0	0	0	0	0	92	100	100	100	85	30	15	69	100
Total tested	13	11	13	13	13	13	13	13	13	13	13	13	13	10	13	13	13

R = resistant, I = intermediate, S = susceptible; AMP = ampicillin, AMC = amoxicillin-clavulanate, TZP = piperacillin-tazobactam, CXM = cefuroxime, CRO = ceftriaxone, CTX = cefotaxime, CAZ = ceftazidime, FEP = cefepime, FOX = cefoxitin, IMP = imipenem, MEM = meropenem, ETP = ertapenem, LVX = levofloxacin, CIP = ciprofloxacin, SXT = trimethoprim-sulphamethoxazole, GEN = gentamicin, AMK = amikacin.

**Table 2:** **MIC distributions for cefotaxime-resistant and piperacillin-tazobactam susceptible** ***E. coli*** **and** ***K. pneumoniae*** **from blood culture isolates tested by Vitek2**

**AMC MIC**	**N (%)**	**FOX MIC**	**N (%)**	**TZP MIC**	**N (%)**
4	19 (21.8)	<=4	60 (72.3)	<=4	65 (70.7)
8	19.0 (21.8)	8	6 (7.2)	8.0	27.0 (29.3)
16	32 (36.8)	16	8 (9.6)	-	-
> = 32	17 (19.5)	32	9 (10.8)	-	-
-	-	> = 64	9 (10.8)	-	-
**Total**	87		83		92

AMC = amoxicillin-clavulanate, FOX = cefoxitin, TZP = piperacillin-tazobactam.

**Table 3:** **Baseline characteristics of patients given definitive monotherapy with a BLBLI or carbapenem**

**Patient characteristic**	**Definitive treatment cohort**		**Total study population**
	**BLBLI (N = 24)**	**Carbapenem (N = 23)**	**N = 91**
Age, median [IQR], years	77 [61–83]	77 [68–83]	75 [62–83]
Female	13 (54%)	12 (52%)	49 (54%)
Hospital acquired	7 (29%)	4 (17%)	20 (22%)
Community acquired	7 (29%)	9 (39%)	42 (46%)
Healthcare associated	10 (42%)	10 (43%)	29 (32%)
CCI, median [IQR]	2 [1-4]	2 [1-5]	2 [1-4]
Pitt score, median [IQR]	1 [0–2]	1 [0–3]	1 [0–2]
APACHEII (if ICU), median [IQR]	26	20	24 [15-28]
ICU admission	2 (8%)	5 (22%)	11 (12.1)
*E. coli*	22 (92%)	17 (74%)	79 (87%)
Source:			
Hepato-biliary	2 (8%)	2 (9%)	12 (13%)
Urinary tract	9 (38%)	13 (57%)	43 (47%)
Neutropenic sepsis	1 (4%)	0 (0%)	4 (4%)
Other/unknown source	12 (50%)	8 (34%)	32 (35%)
Co-morbidity/devices:			
Moderate to severe liver disease	3 (13%)	1 (4%)	7 (8%)
Diabetes without end organ damage	6 (25%)	5 (22%)	21 (23%)
Diabetes with organ damage	4 (17%)	5 (22%)	15 (17%)
Moderate to severe renal disease	4 (17%)	7 (31%)	19 (21%)
Metastatic solid tumour	1 (4%)	1 (4%)	4 (4%)
Leukaemia or lymphoma	1 (4%)	2 (9%)	7 (8%)
Urinary device	5 (21%)	3 (13%)	15 (17%)
Immunosuppressive treatments	2 (8%)	4 (17%)	12 (13%)
Empirical therapy:			
3GC	7 (29%)	6 (26%)	33 (36%)
BLBLI	11 (46%)	5 (22%)	25 (28%)
Carbapenem	0 (0%)	2 (9%)	8 (9%)
Other*	6 (25%)	10 (43%)	25 (27%)
Appropriate empirical therapy	15 (63%)	15 (65%)	50 (55%)

*Including combinations of carbapenem/BLBLI/3CG.

CCI = Charlson Co-morbidity index, IQR = Inter-quartile range, ICU = intensive care unit, 3GC = third-generation cephalosporins, BLBLI = beta-lactam/beta-lactamase inhibitor.

**Table 4:** **Results of Cox regression analyses**

**Outcome**	**Definitive treatment**	**n**	**Crude hazard ratio (95% CI)**	**Adjusted hazard ratio (95% CI)**
30 day mortality	Carbapenem	20	1	1
	BLBLI	21	0.47 (0.09 to 2.59)	0.91 (0.13 to 6.28)*
Resolution of SIRS	Carbapenem	14	1	1
	BLBLI	14	1.19 (0.44 to 3.19)	0.91 (0.32 to 2.59)*
Hospital discharge	Carbapenem	16	1	1
	BLBLI	16	0.74 (0.38 to 1.41)	0.62 (0.27 to 1.42)*

*Adjusted for ICU admission, infecting organism, Pitt score.

## Discussion

In this retrospective observational study, use of a BLBLI as definitive monotherapy for the treatment of cefotaxime non-susceptible *E. coli* or *K. pneumoniae* bloodstream infection was not associated with worse outcome when compared to the use of a carbapenem – often considered standard therapy for such infections [10]. Not only were there no significant differences in 30-day mortality, but there were also no differences in resolution of SIRS criteria over 7 days post bacteraemia. Although event rates were low, there were also no differences in rates of relapsed bacteraemia, *C. difficile* infection or subsequent isolation of a multi-resistant organism. There was a trend towards a longer length of hospital stay in the BLBLI-treated group; although this was not significant (the Cox regression analysis of the length of hospital stay had a power of 55% to detect a hazard ratio of 0.5).

These findings are broadly similar to a prospective study addressing this question from Rodriguez-Bano *et al.* [20] and a meta-analysis that examined studies that reported outcomes for patients treated with BLBLIs for bacteraemia caused by ESBL-producers [28]. In both studies, there were no differences in mortality between patients given BLBLIs when compared with carbapenems for empirical or definitive therapy. As such, these studies would support the concept that BLBLIs, at least, represent a safe carbapenem-sparing option when susceptibility is proven, despite the likely presence of a broad-spectrum beta-lactamase.

Limitations of the study are acknowledged. The total patient cohort was relatively small, especially in the definitive treatment cohort receiving monotherapy with a BLBLI or carbapenem. A significant proportion of patients received either sequential monotherapy with different agents, or various combination therapies, and so were excluded from the analysis thus reducing the sample size. As such, the study was underpowered to detect true differences in infrequent outcomes, particularly for mortality at 30 days. Given the retrospective nature of the study, potential confounders are likely. These might include the propensity to receive a carbapenem (which may occur in patients with a higher risk of mortality) potentially over-estimating the relative efficacy of BLBLIs. We have adjusted for several potential confounding factors where appropriate, however residual confounding from these factors, or confounding from other unmeasured factors, may remain. Although Pitt scores are predictive for mortality in bloodstream infection, additional measures (such as the presence of septic shock or other markers of illness severity at presentation) may have provided further information on potential confounders, but were not available.

Resistance to third-generation cephalosporins, using current EUCAST criteria, is a sensitive but not highly specific marker for ESBL-production. Plasmid-mediated AmpC beta-lactamase acquisition has become widespread in recent years in these species and may provide a similar resistance profile, although are usually also resistant to cefoxitin (in contrast to ESBL-producers). This is of relevance because AmpC enzymes are less effectively inhibited by tazobactam, which could limit clinical efficacy. In this study we were not able to confirm the beta-lactamase types in blood culture isolates. However, amongst *E. coli* and *K. pneumoniae*, 31% and 8% respectively tested non-susceptible (resistant or intermediate) to cefoxitin, suggesting that plasmid-AmpC may occur frequently within *E. coli* in this population. However, ESBLs may themselves cause elevated MICs to cefoxitin, so extrapolation from the antibiogram alone can be misleading. A cefoxitin MIC ≥32 μg/mL has been suggested as a useful screening marker for selecting isolates for confirmatory tests of AmpC production [29]. It is also acknowledged that MIC determination with the Vitek2 instrument, as used in this study, is not a reference method. In common with several other countries, the predominant ESBLs found in Enterobacteriaceae in Singapore are CTX-M types, but plasmid-mediated AmpC-producers are increasingly seen [30,31]. In an ongoing study using whole genome sequencing to characterise third-generation cephalosporin-resistant but piperacillin-tazobactam susceptible *E. coli* or *K. pneumoniae* from BSIs in Singapore, CTX-M-type ESBLs predominated (found in more than 85% of isolates) although plasmid-mediated AmpC (CMY- or DHA-like) β-lactamases were present in around 10% (in-house unpublished data). There has also been a marked increase in the numbers of isolates with acquired carbapenemases in Singapore, [32] highlighting the need to define alternatives to carbapenems where possible.

Further work is needed to definitively test the concept that BLBLIs are, in general, a safe and effective carbapenem-sparing option for the treatment of bloodstream infections caused by third-generation cephalosporin-resistant *E. coli* or *K. pneumoniae*. A large international retrospective observational study has recently reported similar findings to this study [33]. However, studies of this nature are always prone to bias, leaving ongoing uncertainty as to the clinical efficacy of BLBLIs against ESBL-producers. There also remains debate over the effect of MICs that fall in the higher end of the susceptible range, especially for infection outside the urinary tract [34]. A recent retrospective study compared the empirical use of piperacillin-tazobactam with carbapenems for bloodstream infections caused by ESBL-producers, and reported an adjusted risk of death in patients given piperacillin-tazobactam as 1.92 times higher than those given carbapenem therapy (95% CI, 1.07-3.45) [35]. As such, there exists considerable uncertainty in the role of BLBLIs for the treatment of bloodstream infection caused by ESBL-producers. Ideally, such questions should be answered in a randomised controlled trial. Such a study is now underway across several Australasian sites, including Singapore (the ‘MERINO’ trial, registered at www.clinicaltrials.gov; NCT02176122) and aims to be completed by 2018.

## Conclusions

In this retrospective study, comparable outcomes were seen for patients given definitive treatment with BLBLIs or carbapenems for bloodstream infections caused by cefotaxime non-susceptible *E. coli* or *K. pneumoniae* in terms of all-cause mortality, resolution of SIRS, length of stay or bacteraemia relapse. There were also no significant differences in subsequent infection or colonisation with a multi-resistant organism or *C. difficile* infection. However, larger studies adequately powered to detect differences in mortality, preferably in the form of multi-centre randomised trials, are needed before such a strategy can be recommended as standard care.

## Additional file

Additional file 1:
**co-morbidity definitions.**

## Abbreviations

ESBL: Extended-spectrum β-lactamase
BLBLI: β-lactam/β-lactamase inhibitor
BSI: Bloodstream infection
SIRS: Systemic inflammatory response syndrome
EUCAST: European Committee on Antimicrobial Susceptibility Testing
PICC: Peripherally inserted central catheter
TNF: Tumour necrosis factor
ICU: Intensive care unit
APACHE: Acute Physiology and Chronic Health Evaluation
HAI: Hospital-acquired infection
MALDI-TOF: Matrix Assisted Laser Desorption Ionization Time-of-Flight
CCI: Charlson co-morbidity index
MIC: Minimum inhibitory concentration
HR: Hazard ratio
