# Supplementary material for: Comparable outcomes for β-lactam/β-lactamase inhibitor combinations and carbapenems in definitive treatment of bloodstream infections caused by cefotaxime-resistant Escherichia coli or Klebsiella pneumoniae
Source: Antimicrob Resist Infect Control. 2015 May 1;4:14. doi: 10.1186/s13756-015-0055-6 (PMC4414382; doi:10.1186/s13756-015-0055-6)
Supplement: Additional file 1: — co-morbidity definitions. [file 13756_2015_55_MOESM1_ESM.docx]

**Appendix** – Comorbidity definitions (adapted from Charlson *et al*[[33](#_ENREF_33)])

- Myocardial infarction: patients with at least one definite or probable myocardial infarction requiring hospitalization; with ECG and/or troponin changes. ECG changes alone were not designated as having had an infarction.
- Congestive heart failure: patients with exertional or paroxysmal nocturnal dyspnoea and response to diuretics or afterload reducing agents.
- Peripheral vascular disease: patients with intermittent claudication or those who had bypass or angioplasty for arterial insufficiency, gangrene or acute arterial insufficiency and untreated thoracic or abdominal aneurysm.
- Cerebrovascular disease: patients with a history of ischaemic or haemorrhagic stroke with minor or no residual deficits
- Hemiplegia: as a result of a cerebrovascular accident or other conditions
- Dementia: patients with chronic cognitive deficit
- Chronic pulmonary disease: chronic pulmonary syndrome leading to breathlessness or intermittent exacerbation or need for chronic oxygen therapy, or those with chronic CO_2_ retention and / or baseline PaO_2_ <50mmHg.
- Diabetes with end organ damage, e.g. retinopathy, neuropathy or nephropathy.
- Diabetes without end organ damage: all other patients with diabetes mellitus with no evidence of end organ damage
- Severe renal disease: patients on dialysis or with a renal transplant or those with uraemia.
- Severe liver disease: patients with cirrhosis, portal hypertension and a history of variceal bleeding
- Moderate liver disease: cirrhosis with portal hypertension, but without history of bleeding
- Mild liver disease: of cirrhosis without portal hypertension or chronic hepatitis
- Peptic ulcer disease: patients who have required treatment for endoscopically confirmed peptic ulcer disease,
- HIV/AIDS: patients with confirmed HIV infection, including with AIDS defining illness or anti-retroviral therapy
- Lymphoma: includes Hodgkins disease, Waldenstrom’s macroglobulinemia, multiple myeloma and other lymphomas
- Leukaemia: patients with acute and chronic myeloid leukaemia, acute and chronic lymphocytic leukaemia, and polycythaemia vera
- Metastatic cancer: patients with metastatic solid tumours, including breast, lung, colon and other tumours
- Connective tissue disease: history of rheumatoid arthritis or other seronegative arthritis, vasculitis, systemic lupus erythematosus, dermato/polymyositis or scleroderma or any other autoimmune disease affecting connective tissue
